# Integrated differential transcriptome maps of Acute Megakaryoblastic Leukemia (AMKL) in children with or without Down Syndrome (DS)

**DOI:** 10.1186/s12920-014-0063-z

**Published:** 2014-12-05

**Authors:** Maria Chiara Pelleri, Allison Piovesan, Maria Caracausi, Anna Concetta Berardi, Lorenza Vitale, Pierluigi Strippoli

**Affiliations:** Department of Experimental, Diagnostic and Specialty Medicine (DIMES), Unit of Histology, Embryology and Applied Biology, University of Bologna, Via Belmeloro 8, 40126 Bologna, BO Italy; Research Laboratory Stem Cells, U.O.C. Immunohematology-Transfusion Medicine and Laboratory of Hematology, Santo Spirito’s Hospital, Via del Circuito, 65100 Pescara, Italy; Interdepartmental Center for Cancer Research Giorgio Prodi (CIRC), S. Orsola-Malpighi Hospital, University of Bologna, Via Massarenti 9, 40138 Bologna, BO Italy

**Keywords:** Down Syndrome (Trisomy 21), Acute Megakaryoblastic Leukemia (AMKL), Transient Myeloproliferative Disorder (TMD), Megakaryocyte (MK), Gene expression profile, Integrated transcriptome map

## Abstract

**Background:**

The incidence of Acute Megakaryoblastic Leukemia (AMKL) is 500-fold higher in children with Down Syndrome (DS) compared with non-DS children, but the relevance of trisomy 21 as a specific background of AMKL in DS is still an open issue. Several Authors have determined gene expression profiles by microarray analysis in DS and/or non-DS AMKL. Due to the rarity of AMKL, these studies were typically limited to a small group of samples.

**Methods:**

We generated integrated quantitative transcriptome maps by systematic meta-analysis from any available gene expression profile dataset related to AMKL in pediatric age. This task has been accomplished using a tool recently described by us for the generation and the analysis of quantitative transcriptome maps, TRAM (Transcriptome Mapper), which allows effective integration of data obtained from different experimenters, experimental platforms and data sources. This allowed us to explore gene expression changes involved in transition from normal megakaryocytes (MK, n=19) to DS (n=43) or non-DS (n=45) AMKL blasts, including the analysis of Transient Myeloproliferative Disorder (TMD, n=20), a pre-leukemia condition.

**Results:**

We propose a biological model of the transcriptome depicting progressive changes from MK to TMD and then to DS AMKL. The data indicate the repression of genes involved in MK differentiation, in particular the cluster on chromosome 4 including *PF4* (platelet factor 4) and *PPBP* (pro-platelet basic protein); the gene for the mitogen-activated protein kinase *MAP3K10* and the thrombopoietin receptor gene *MPL*. Moreover, comparing both DS and non-DS AMKL with MK, we identified three potential clinical markers of progression to AMKL: *TMEM241* (transmembrane protein 241) was the most over-expressed single gene, while *APOC2* (apolipoprotein C-II) and *ZNF587B* (zinc finger protein 587B) appear to be the most discriminant markers of progression, specifically to DS AMKL. Finally, the chromosome 21 (chr21) genes resulted to be the most over-expressed in DS and non-DS AMKL, as well as in TMD, pointing out a key role of chr21 genes in differentiating AMKL from MK.

**Conclusions:**

Our study presents an integrated original model of the DS AMLK transcriptome, providing the identification of genes relevant for its pathophysiology which can potentially be new clinical markers.

**Electronic supplementary material:**

The online version of this article (doi:10.1186/s12920-014-0063-z) contains supplementary material, which is available to authorized users.

## Background

Trisomy for human chromosome 21 (chr21) is the most frequent live-born aneuploidy and is the cause of Down syndrome (DS), whose main symptoms include intellectual disability, cardiovascular defects and craniofacial dysmorphisms [1]. The DS phenotype is thought to be associated with an altered expression of the genes located on chr21 [2-7]. Basic research on DS is now rapidly accelerating, and there is the possibility that the results will be beneficial for individuals with DS [8].

Several studies have shown that individuals with DS have a specific cancer risk pattern, or tumor profile: their risk of developing leukemia and testicular cancer is much higher than age-matched controls, while women with DS almost never develop breast cancer [9,10]. In particular, children with DS show an increased prevalence of acute leukemia, both lymphoid (ALL) and myeloid (AML), with relative risk ranging from 10 to 20 times higher than the normal population [11,12]. In nearly half of the cases, these childhood leukemias are classified as megakaryoblastic leukemia (AMKL), a relatively rare subtype of AML also known as AML M7, according to French–American–British (FAB) classification*,* whose incidence increases by 500-fold in children with DS by the age of 4 years as compared to the chromosomally normal population (reviewed in [13]). This observation strongly suggests that trisomy 21 directly contributes to the neoplastic transformation of hematopoietic cells, in particular in the megakaryocyte lineage cells. Interestingly, acute leukemia cells harboring megakaryocyte markers and presenting in subjects without DS may show trisomy 21 [14]. We also described a cell line derived from blast cells of a patient with type M2 AML which has trisomy 21 and megakaryocyte features [15]. More recently, mutations of the gene encoding for the transcription factor GATA1 have been shown to cooperate with trisomy 21 in initiating megakaryoblastic proliferation in nearly all DS AMKL cases while they are absent in non-DS AMKL [13,16]. *GATA1* mutations in DS cells give rise to a short, truncated form of GATA1 (GATA1s) transcription factor that, in this form, is not able to establish normal interactions with other gene regulators [17].

Transient myeloproliferative disorder (TMD) is a clonal pre-leukemia condition, occurring in 10% of children with DS during the neonatal period, presenting at a median age of 3-7 days with accumulation of immature megakaryoblasts [13]. TMD cases usually resolve spontaneosuly, but DS AMKL may develop within 1-4 years in 20-30% of these children. AMKL may develop in non-DS children, usually at an higher age in comparison to DS subjects (median 8 vs. 1.8 years, respectively) and in absence of a trisomy 21 background. Cytogenetic abormalities described in non-DS AMKL cells include trisomy 8 and 1 and monosomy 7 [13].

An open issue is the relevance of trisomy 21 as a specific background for the higher incidence of AMKL in DS. A few previous studies have used gene expression profiling by microarray analysis in order to identify specific transcriptome alterations in DS and/or non-DS AMKL, as well as in TMD [17-24]. Due to the rarity of AMKL, these works often analyze a small number of cases, using a variety of experimental platforms. Results were consequently affected by a small grade of comparability.

One of the first goals of this work was to perform a systematic meta-analysis using any available gene expression profile dataset related to AMKL in pediatric age in order to produce a differential transcriptome map between DS and non-DS related AMKL. This task has been accomplished using a tool recently described by us for the generation and the analysis of quantitative transcriptome maps, TRAM (Transcriptome Mapper) [25], which allows effective integration of data obtained from different experimenters, experimental platforms and data sources. The comparison of 43 DS AMKL samples with 45 non-DS AMKL samples represents the largest study on the subject, highlighting the relevance of trisomy 21 in the development of AMKL in comparison with AMKL originating from non-trisomic cells. Results show significant over- or under-expression of distinct chromosomal segments and of single key genes in the whole genome, as well as on chr21, adding new knowledge compared with that produced by the single works from which the data were originally obtained. In addition, each considered type of leukemia was compared with the expression profile of TMD cells and normal human megakaryoblast/megakaryocyte cells (MK), allowing the building of a model for the disorder in differentiation process that lead to DS and non-DS AMKL. Comparisons with cord blood-derived MK cells (CB MK) have also been performed, due to the fact that leukemias in infants or young children originate from fetal hematopoietic cells [17,18,26,27] and the progenitor cells (fetal/neonatal MKP) are present in the cord blood (CB) [28,29].

For each cell type investigated, reference expression data for about 17,000-26,000 mapped sequences have been generated and validated through a sample comparison with known data. The biological and clinical significance of these data is discussed.

## Methods

### Literature search

A systematic biomedical literature search was performed up to January 2013 in order to identify articles related to global gene expression profile experiments in AMKL patients (DS AMKL, non-DS AMKL and TMD conditions). A general search using the commonly used acronym "AMKL" retrieved 157 articles.

The MeSH term "Leukemia, Megakaryoblastic, Acute" was also used for a PubMed search in the expression: "Leukemia, Megakaryoblastic, Acute"[Mesh] AND ("Gene Expression Profiling"[MeSH] OR "Oligonucleotide Array Sequence Analysis"[Mesh] OR "Microarray Analysis"[Mesh] OR microarray* OR "Expression profile" OR SAGE).

### Database search

Gene Expression Omnibus (GEO) [30] functional genomics repository was searched for: (AMKL[All Fields] OR (AML[All Fields] AND M7[All Fields])) AND "Homo sapiens"[Organism]. A more general search using the expression "Down Syndrome"[MeSH] AND "Homo sapiens" [Organism] was also used.

ArrayExpress database [31] of functional genomics experiments was searched for the terms: ''AMKL'', "Megakaryoblastic", "AML M7".

In order to obtain gene expression profile datasets for normal human MK cells, in addition to the 9 used in the original description of the TRAM software [25], we searched GEO for the expression ("Megakaryocytes" [Mesh] OR Megakaryoblast*) AND "Homo sapiens" [ORGANISM]. The ArrayExpress database was searched for the expressions "Megakaryocyte", "Megakaryocytic", "Megakaryoblast", "MK".

The searches were performed up to January 2013.

### Dataset selection

The inclusion criteria of datasets in the analysis were: availability of the raw or pre-processed data; pediatric age of the subject from whom the sample was obtained; diagnosis of DS or non-DS AMKL or TMD.

Exclusion criteria were: exon arrays (hampering data elaboration by TRAM due to exceedingly high number of data rows) or platforms using probes split into several distinct arrays for each sample (hampering intra-sample normalization); lack of identifiers corresponding to those found in the GEO sample records (GSM) or ArrayExpress sample records; platforms assaying an atypical number of genes (i.e. <5.000 or >60.000); cell line derived data; specific subtype of non-DS AMKL, e.g. t(1;22); trisomy 21 in non-DS AMKL samples.

Normal MK samples were considered for the analysis when fulfilling these criteria: late MK colonies (10-14 days) or MK sorted cells, obtained from peripheral blood (PB), bone marrow (BM) or cord blood (CB). MK cultured for less than 10 days or Colony Forming Unit-Megakaryocytic (CFU-MK) were excluded.

In order to obtain a quantitative transcriptome map, values from each dataset were linearized when provided as logarithms. In some cases we used raw files (e.g. File CEL) to be converted into pre-processed data, using the software "Alt Analyze" [32].

### TRAM (transcriptome mapper) analysis

TRAM (Transcriptome Mapper) software [25] allows the import of gene expression data recorded in the NCBI (National Center for Biotechnology Information) GEO and EBI (European Bioinformatics Institute) ArrayExpress databases in tab-delimited text format. It also allows the integration of all data by decoding probe set identifiers to gene symbols via UniGene data parsing [33], normalizing data from multiple platforms using intra-sample and inter-sample normalization (scaled quantile normalization) [34], creating graphical representation of gene expression profile through two ways, "Map" and "Cluster" mode, and determining the statistical significance of results. Moreover, TRAM allows to compare two biological conditions identifying critical genomic regions and genes with significant differential expressions.

We created a directory (folder) for each condition, containing all the sample datasets related to the same source and selected for the study: DS AMKL (pool 'A'); non-DS AMKL (pool 'B'); TMD (pool 'C'); normal MK (pool 'D'); normal CB MK (pool 'E').

We ran the whole set of analyses permitted by TRAM (in both "Map" and "Cluster" mode, although we focused on the "Map" mode) using default parameters as described [25]. We used an updated version of TRAM including enhanced resolution of gene identifiers and updated UniGene and Entrez Gene databases (TRAM 1.1, June 2013), in comparison with the original 2011 version [25]. When the gene location cytoband was not available in the Gene database [35], it was manually derived from UCSC Genome Browser [36]. TRAM is freely available at http://apollo11.isto.unibo.it/software. Briefly, gene expression values were assigned to individual loci via UniGene, intra-sample normalized as percentage of the mean value and inter-sample normalized by scaled quantile. The value for each locus, in each biological condition, is the mean value of all available values for that locus. The genome wide gene expression median value was used in order to determine percentiles of expression for each gene.

Using the "Map" mode graphical representation we searched for over/under-expressed genome segments, which have a window size of 500,000 bp and a shift of 250,000 bp. The expression value for each genomic segment is the mean of the expression values of the loci included in that segment. A segment is defined over/under-expressed if it has an expression value which is significantly different between two conditions analyzed, and contains at least 3 individually over/under-expressed genes, e.g. genes which have expression values within the highest and the lowest 2.5th percentile. Significance of the over/under-expression for single genes was determined by running TRAM in "Map" mode with a segment window of 12,500 bp. This window size corresponds to about a quarter of the mean length of a gene, so the significant over/under-expression of a segment almost always corresponds with that of a single gene. A segment or a gene was considered to be statistically significantly over- or under-expressed for q < 0.05, where q is the p-value obtained by the method of hypergeometric distribution [25] and corrected for multiple comparison. When the segment window contains more than one gene, the significance is maintained if the expression value of the over/under-expressed gene prevails over the others.

For the creation of the maps, TRAM software does not consider probes where the expression values is not available, assuming that an expression level has not been measured. Furthermore, it gives 95% of the minimum positive value present in a sample to those expression values equal to or lower than "0", in order to obtain meaningful numbers when we need to obtain a ratio between values in pool 'A' and pool 'B'. Assuming that in these cases an expression level is too low to be detected under the experimental conditions used, this transformation is useful to highlight differential gene expression. Finally, we considered the most over- or under-expressed genes among the genes associated with at least 5 data points.

At chromosomal level, we calculated (in the TRAM "chr" table) the median expression ratio for all the genes located in the same chromosome.

### Other analysis

FuncAssociate analysis [37] was used to obtain Gene Ontology attributes in order to functionally characterize large sets of genes derived from the TRAM analysis.

## Results

### Literature search

A general search using the acronym ''AMKL'' retrieved 157 articles, 6 of them describe gene expression profiling experiments [17,18,20-23].

No additional pertinent item was retrieved using the expression described in the Methods section and including the MeSH Term "Leukemia, Megakaryoblastic, Acute".

### Database search

The Gene Expression Omnibus (GEO) [30] search allowed the retrievement of three additional works describing data possibly useful for meta-analysis [19,24,38]. The lack of inclusion of these works in the literature search was due to failure of using the ''AMKL'' acronym and assigning the MeSH Term "Leukemia, Megakaryoblastic, Acute" during the PubMed indexing process (the more general term "Leukemia, Myeloid, Acute" was used).

The more general search using the expression "Down Syndrome"[MeSH] AND "Homo sapiens"[Organism] allowed the addition of one further work [39]. This work analyzed several types of AML samples and did not explicitly mention AMKL or AML M7 in both PubMed and GEO databases.

No further pertinent works related to AMKL were identified by ArrayExpress database [31] search.

Several datasets for normal MK cells global gene expression profile fulfilling the selection criteria were obtained from the works [40] (GEO, 7 samples) and [41] (ArrayExpress, 1 sample), in addition to the 4 sample series identified in the first report of the TRAM software [25] and obtained from different works [42-45], for a total of 19 datasets related to human normal MK cells.

### Dataset building

Of the 10 works related to DS or non-DS AMKL retrieved as above described, 7 were considered for the meta-analysis (Table 1). It was not possible to obtain raw data from the Authors of [20], while the only sample of AML M7 described in [38] was related to "Leukemic Stem Cells" cell type and the two AML M7 reported by Tomasson et al. [39] were obtained from elderly patients. Raw data from [23] were kindly provided by Drs. Jeffrey Taub and Yubin Ge.

**Table 1:** **Main features of the samples used in TRAM analyses**

**Study ID**	**Sample ID**	**Sample type**	**Platform**	**Microarray**	**Spots**	**References**
**Pool** **'A'** **(n=43) - DS AMKL** (25,955 mapped loci following analysis by TRAM)						
A1…A3 (n=3)	GSM491372…4	BM Sorted leukemic blasts	GPL570	Affymetrix U133 Plus 2.0	54,675	[18]
A4…A25 (n=22)	GSM94245, GSM94272…92	BM or PB	GPL96	Affymetrix U133A	22,283	[22]
A26…A31 (n=6)	GSM417985…90	BM or PB Sorted leukemic blasts	GPL570	Affymetrix U133 Plus 2.0	54,675	[17]
A32…A38 (n=7)	E-MEXP-72*	BMMC or PBMC	A-AFFY-33.adf.txt*	Affymetrix U133A	22,283	[21]
A39…A43 (n=5)	/	BMMC or PBMC	GPL96	Affymetrix U133A	22,283	[23]
**Pool 'B' (n=45) - non-DS AMKL** (26,045 mapped loci following analysis by TRAM)						
B1-B2 (n=2)	GSM491370-1	BM Sorted leukemic blasts	GPL570	Affymetrix U133 Plus 2.0	54,675	[18]
B3…B23 (n=21)	GSM94221-4-5, GSM94227…32, GSM94234-5-7-8, GSM94240-2-3-8, GSM94256-9, GSM94261-2	BM or PB	GPL96	Affymetrix U133A	22,283	[22]
B24…B28 (n=5)	GSM39832-5, GSM39842-4, GSM39863	PBMC or BMMC	GPL8300	Affymetrix U95 Version 2	12,625	[19]
B29…B35 (n=7)	GSM361502…4, GSM361506…8, GSM361510	BM or PB	GPL96	Affymetrix U133A	22,283	[24]
B36…B40 (n=5)	GSM417991…5	BM or PB Sorted leukemic blasts	GPL570	Affymetrix U133 Plus 2.0	54,675	[18]
B41…B45 (n=5)	/	BMMC or PBMC	GPL96	Affymetrix U133A	22,283	[23]
**Pool 'C' (n=20) - TMD** (25,955 mapped loci following analysis by TRAM)						
C1…C3 (n=3)	GSM491375…7	BM Sorted leukemic blasts	GPL570	Affymetrix U133 Plus 2.0	54,675	[18]
C4…C11 (n=8)	GSM94293…9, GSM94300	BM or PB	GPL96	Affymetrix U133A	22,283	[22]
C12…C20 (n=9)	E-MEXP-72*	BMMC or PBMC	A-AFFY-33.adf.txt*	Affymetrix U133A	22,283	[21]
**Pool 'D' (n=19) - MK** (26,372 mapped loci following analysis by TRAM)						
D1-D2 (n=2)	GSM321577-8	MK (BM) (subj = pool)	GPL96	Affymetrix U133A	22,283	[45]
D3…D6 (n=4)	GSM112277-8, GSM112291-2	MK (PB) (subj = 1, rep. 1)	GPL887	Agilent 1A	22,575	[44]
D7-D8 (n=2)	GSM15648, GSM8649	MK (BM) (subj = 6)	GPL96	Affymetrix U133A	22,283	[42]
D9…D11 (n=3)	GSM88014-22-34	MK (PB) (subj = 1)	GPL887	Agilent 1A	22,575	[43]
D12…D18 (n=7)	GSM609746…52	CB	GPL4685	Affymetrix HT-HG_U133A	22,944	[40]
D19 (n=1)	E-MEXP-2146*	CB	A-AFFY-44.adf.txt	Affymetrix U133 Plus 2.0	54,675	[41]
**Pool 'E' = D12…D19 (n=8) - CB MK** (25,577 mapped loci following analysis by TRAM)						

Samples selected for the meta-analysis of gene expression profiles in DS AMKL (pool 'A'), non-DS AMKL (pool 'B'), TMD (pool 'C'), and megakaryocytic cells (pool 'D' and 'E'). All Sample IDs and Platforms IDs are related to GEO database, other than codes marked with * (ArrayExpress database). **Sample type:** BM, bone marrow; PB, peripheral blood; BMMC, bone marrow mononuclear cells; PBMC, peripheral blood mononuclear cells; MK, megakaryocytic/megakaryoblast cells, obtained by in vitro differentiation of CD34+ cells; CD34+, undifferentiated CD34+ cells; BM, CB or PB: CD34+ cells derived from bone marrow, cord blood or peripheral blood, respectively. subj = number of subjects from which the sample was derived (in some cases, where subj = pool, the exact number of subjects included in a pool was not available). rep. = biological replicate. **Microarray:** U133A: Affymetrix Human Genome U133A Array; 1A: Agilent-012097 Human 1A Microarray (V2) G4110B; 22k A: Agilent Human oligo 22k A; HG-Focus: Affymetrix Human HG-Focus Target Array.

Details about Sample identifiers and main sample features are listed in Additional file 1 (available at: http://apollo11.isto.unibo.it/suppl).

At the end, DS AMKL sample pool 'A' included 43 datasets, while non-DS AMKL sample pool 'B' was composed of 45 datasets. A TMD dataset pool 'C' was constructed starting from 20 samples described in some of the DS AMKL related articles [18,21,22]. Age and sex data were available for 29 out of 43 DS AMKL patients (mean age: 20 months; 11 males and 18 females), for 26 out of 45 non-DS AMKL patients (mean age: 19 months; 19 males and 7 females) and for 9 out of 20 TMD patients (mean age: 8 days; 7 males and 2 females). *GATA1* mutations giving rise to GATA1s were present in all DS AMKL and TMD samples, and not in non-DS AMKL samples, considering all samples for which this information was provided. Sample identifiers and main sample features are listed in Table 1 and Additional file 1 (available at: http://apollo11.isto.unibo.it/suppl).

Two pools were constructed from the normal MK related dataset selected: pool 'D' included all available MK samples, while pool 'E' was a subset including only CB-derived MK cells (Table 1 and Additional file 1).

### Transcriptome differential maps

Datasets were loaded into TRAM and analyzed obtaining 8 transcriptome maps: DS AMKL (pool 'A') vs. non-DS AMKL (pool 'B'); DS AMKL (pool 'A') vs. normal MK (pool 'D'); non-DS AMKL (pool 'B') vs. normal MK (pool 'D'); DS AMKL (pool 'A') vs. normal CB MK (pool 'E') cells; non-DS AMKL (pool 'B') vs. normal CB MK (pool 'E'); DS AMKL (pool 'A') vs. TMD (pool 'C'); TMD (pool 'C') vs. normal MK (pool 'D'); TMD (pool 'C') vs. normal CB MK (pool 'E').

For each comparison between two cell types by TRAM, we describe below or in the corresponding Figures or Tables the total of data points analyzed for each cell type, i.e. gene expression values for all human mapped loci following intra- and inter-sample normalization [25]; the number of loci for which the comparison between the two conditions was possible due to the presence of values for those loci in both sample pools considered; the number and the gene content of each genomic segment containing at least three over- or under-expressed genes and found to be statistically significantly over- or under-expressed in the comparison between the two tissues. Each genomic segment was identified among the 12,373 segments generated using the default window of 500,000 bp with a sliding window of 250,000 bp and following removal of overlapping segments with similar gene content. When the results were reported for the over/under-expressed single genes, we considered only the genes associated at at least 5 data points.

The description of the gene name corresponding to all gene symbols cited here in the text, Figures or Tables is given in the Additional file 2. We performed a PubMed search for the most relevant over- or under-expressed genes using gene symbol or gene description along with MeSH terms related to MK or MK progenitor cells, thrombopoiesis, AMKL, platelets.

Detailed results for each map are provided below, and are also available at: http://apollo11.isto.unibo.it/suppl.

The absolute (not differential) expression values and maps for each cell type (not compared to another cell type) are also available in the complete sets of results at http://apollo11.isto.unibo.it/suppl, but are not discussed here because they include typical housekeeping genes whose over-expression is no longer evident when compensated by the corresponding housekeeping genes in the compared cell type.

### Transcriptome map comparison of DS AMKL vs. non-DS AMKL

**Figure 1:**
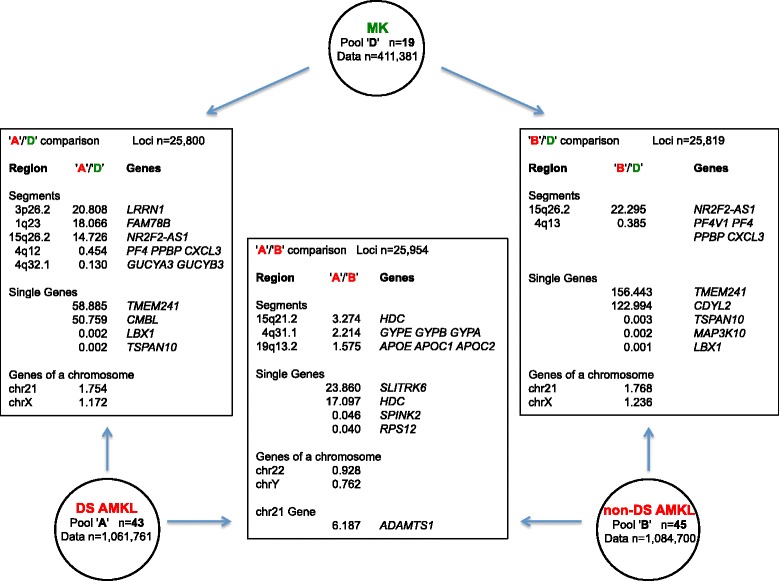
**Main results of DS AMKL vs. MK, DS AMKL vs. non-DS AMK, non-DS AMKL vs. MK comparisons.** For each comparison the number of loci analyzed, the most over- or under-expressed segments and single genes, and the highest and lowest median expression ratios for all the genes located in the same chromosome are indicated.

We first analyzed regional differential expression of pool 'A' (43 DS AMKL samples) versus pool 'B' (45 non-DS AMKL samples) (Table 1). A total of 1,061,761 data points from the pool 'A' and 1,084,700 data points from the pool 'B' were included in the analysis. An 'A'/'B' ratio value was determinable for 25,954 loci having values both in 'A' and 'B' pools (Additional file 3). The main results are shown in Figure 1. Results obtained by the analysis included 3 significantly non-overlapped over-expressed segments (Table 2a). The highest expression ratio between DS AMKL and non-DS AMKL (3.27) was observed in a segment on chromosome 15 (15q21.2), including the known gene *HDC* (encoding for histidine decarboxylase, which converts L-histidine to histamine). The second segment with the highest expression was located on chromosome 4 (4q31.1) and contained over-expressed genes such as *GYPE, GYPB* and *GYPA*, encoding for glycophorin E, B and A (MNS blood group) respectively. The third over-expressed segment spans the cluster of apolipoprotein encoding genes on chromosome 19 (19q13.2).

**Table 2:** **Genomic segments significantly over- or under-expressed**

**a) DS AMKL vs. non-DS AMKL**					
**Chr and ** **location** ^**a**^	**Segment ** **start** ^**b**^	**Segment ** **end** ^**b**^	**'A'/'B' ratio**	**q-value**	**Genes in the segment** ^**c**^
**Over-expressed segments**					
chr15 15q21.2	50,250,001	50,750,000	3.27	0.00233	*ATP8B4- SLC27A2+* ***HDC+*** *GABPB1+ FLJ10038+ GABPB1-AS1+* **Hs.656448+ Hs.660869+** ^**d**^ *USP8+*
chr4 4q31.1	144,750,001	145,250,000	2.21	0.00024	***GYPE+*** Hs.658686- ***GYPB*** **+** ***GYPA*** **+**
chr19 19q13.2	45,000,001	45,500,000	1.58	0.00461	*ZNF180- PVR+ CEACAM19- BCL3+ CBLC+ BCAM+ PVRL2-* Hs.666142- *TOMM40+* ***APOE+ APOC1*** **+** *APOC4+* ***APOC2*** **+ ** *CLPTM1+*
**b) DS AMKL vs. MK**					
**Chr and location**	**Segment start**	**Segment end**	**'A'/'D' ratio**	**q-value**	**Genes in the segment**
**Over-expressed segments**					
chr3 p26.2	3,500,001	4,000,000	20.81	0.00006	**Hs.241414+** Hs.587205+ ***LRRN1*** **+ Hs.128128+**
chr1 1q23	166,000,001	166,500,000	18.07	0.00004	**Hs.22930+** ***FAM78B+*** **Hs.662048+**
chr15 15q26.2	96,750,001	97,250,000	14.73	0.00001	**Hs.677040+ Hs.661950+** ***NR2F2-AS1*** **+** *NR2F2-* **Hs.592015+**
**Under-expressed segments**					
chr4 4q12-q21	74,750,001	75,250,000	0.45	0.00079	***PF4*** **-** ***PPBP*** **-** *CXCL5-* ***CXCL3*** **-** *CXCL2- MTHFD2L-* Hs.662627- *EREG-*
chr4 4q32.1	156,250,001	156,750,000	0.13	0.00012	*MAP9-* ***GUCY1A3*** **- Hs.612374-** ***GUCY1B3*** **-**
**c) Non-DS AMKL vs. MK**					
**Chr and location**	**Segment start**	**Segment end**	**'B'/'D' ratio**	**q-value**	**Genes in the segment**
**Over-expressed segments**					
chr15 15q26.2	96,750,001	97,250,000	22.30	<0.00001	**Hs.677040+ Hs.661950+** ***NR2F2-AS1*** **+** *NR2F2-* **Hs.592015+**
**Under-expressed segments**					
chr4 4q13-q21	74,500,001	75,000,000	0.39	0.00009	*IL8- CXCL6-* ***PF4V1*** **-** *CXCL1-* ***PF4*** **-** ***PPBP*** **-** *CXCL5-* ***CXCL3*** **-** *CXCL2-*

Data refer to the following comparisons: **a)** DS AMKL (pool 'A') vs. non-DS AMKL (pool 'B'); **b)** DS AMKL (pool 'A') vs. MK (pool 'D'); **c)** non-DS AMKL (pool 'B') vs. MK (pool 'D'). Analysis was performed using default parameters (see Methods section). Segments are sorted by decreasing 'A'/'B' ratio in **a)**, 'A'/'D' ratio in **b)**, 'B'/'D' ratio in **c)**. In the "Map" mode, TRAM displays UniGene EST clusters (with the prefix "Hs." in the case of *Homo sapiens*) only if they have an expression value. Some segments are not shown for simplicity because they are over-lapping with those highlighted in one of the listed regions. The complete results for these models are available as on line additional material.

^a^Chr: chromosome. The segment location cytoband was derived from that of the first mapped gene within the segment.

^b^Segment Start/End: chromosomal coordinates for each segment.

^c^Bold and '+': over-expressed gene; bold and '-': under-expressed gene; '+' or '-': gene expression value higher or lower than the median value, respectively.

^d^This UniGene cluster contains EST with at least one Alu sequence, according to Repeat Masker [46]. For this reason, it can not be excluded that its over-expression is related to unspecific hybridization by Alu-containg probes.

At single gene level, a fold increase higher than 5 was observed in all of the first 20 genes with the greatest expression ratios of DS AMKL vs. non-DS AMKL samples (Table 3a and Additional file 3). In particular, a 24-fold increase was observed for *SLITRK6* gene, encoding for a membrane protein to date described as similar to receptor for BDNF (brain-derived neurotrophic factor) and predominantly expressed in neural tissues. Among the genes with the lower 'A'/'B' expression ratios a 169.5-fold decrease was observed for a UniGene EST cluster, Hs.355689.

**Table 3:** **List of the five most over- or under-expressed genes (all significantly, with q < 0.05)**

**a) DS AMKL vs. non-DS AMKL**								
**Gene**	**Value 'A'**	**Value 'B'**	**'A'/'B' ratio**	**Location**	**Data points**		**SD**	**SD**
					**'A'**	**'B'**	**'A'**	**'B'**
**Over-expressed genes**								
*SLITRK6*	465.5	19.5	23.860	13q31.1	27	21	113.0	79.6
*HDC*	1,119.4	65.5	17.097	15q21-q22	36	45	98.8	249.4
*ZNF587B*	615.0	39.3	15.643	19q13.43^a^	43	40	427.6	32.2
*SOSTDC1*	111.7	8.6	12.957	7p21.1	43	45	176.5	108.4
LOC100287628*	1,381.3	107.7	12.820	16p13.2	9	7	80.2	56.8
**Under-expressed genes**								
Hs.587427*	19.8	407.8	0.049	7p15.2	18	14	28.9	353.3
*SPINK2*	13.1	285.1	0.046	4q12	36	45	81.8	115.0
Hs.602709*	15.8	370.2	0.043	11q13.2^a^	9	7	33.1	256.9
*RPS12*	20.7	519.1	0.040	6q23.2	43	50	113.7	213.0
Hs.355689*	3.7	624.4	0.006	18q11.2^a^	9	7	47.3	168.4
**b) DS AMKL vs. MK**								
**Gene**	**Value 'A'**	**Value 'D'**	**'A'/'D' ratio**	**Location**	**Data points**		**SD**	**SD**
					**'A'**	**'D'**	**'A'**	**'D'**
**Over-expressed genes**								
*TMEM241*	1,036.6	17.6	58.885	18q11.2	18	8	57.7	115.8
*CMBL*	592.4	11.7	50.759	5p15.2	27	8	142.0	30.1
*IFI27*	467.8	11.6	40.474	14q32	36	82	90.0	27.6
*CSRNP1*	640.6	16.8	38.023	3p22	9	8	41.3	138.3
*PTGS2*	2,205.0	59.2	37.276	1q25.2 q25.3	63	20	156.4	131.6
APOC2* ^b^	431.7	11.7	37.044	19q13.2	54	28	81.4	41.6
*SLITRK6* ^b^	465.5	13.3	35.057	13q31.1	27	10	113.0	95.6
**Under-expressed genes**								
*MAP3K10*	5.4	2,352.3	0.002	19q13.2	36	19	47.4	135.1
*DRD4*	5.0	2,327.6	0.002	11p15.5	36	19	51.3	135.2
*DLGAP3*	4.8	2,297.9	0.002	1p35.3-p34.1	9	7	40.8	37.6
*LBX1*	2.7	1,358.5	0.002	10q24	36	19	46.4	152.6
TSPAN10*	7.9	4,674.7	0.002	17q25.3	9	8	55.0	46.0
**c) Non-DS AMKL vs. MK**								
**Gene**	**Value 'B'**	**Value 'D'**	**'B'/'D' ratio**	**Location**	**Data points**		**SD**	**SD**
					**'B'**	**'D'**	**'B'**	**'D'**
**Over-expressed genes**								
*TMEM241*	2,754.1	17.6	156.443	18q11.2	14	8	89.9	115.8
*CDYL2*	1,613.5	13.1	122.994	16q23.2	7	8	128.9	69.8
*MPV17L*	1,857.8	24.4	76.280	16p13.11	7	8	119.5	65.0
*ATP6V0D2*	836.7	11.9	70.488	8q21.3^a^	21	10	169.4	82.9
*CMBL*	757.9	11.7	64.938	5p15.2	21	8	176.2	30.1
**Under-expressed genes**								
*ILDR1*	13.7	4,333.8	0.003	3q13.33	14	9	98.4	63.3
*HIST3H3*	3.9	1,313.0	0.003	1q42	40	19	78.0	137.9
TSPAN10*	12.1	4,674.7	0.003	17q25.3	7	8	88.1	46.0
*MAP3K10*	3.9	2,352.3	0.002	19q13.2	45	19	40.1	135.1
*LBX1*	2.0	1,358.5	0.001	10q24	45	19	85.7	152.6

Data refer to the following comparisons: **a)** DS AMKL (pool 'A') vs. non-DS AMKL (pool 'B'); **b)** DS AMKL (pool 'A') vs. MK (pool 'D'); **c)** non-DS AMKL (pool 'B') vs. MK (pool 'D'). Value: mean gene expression value normalized across all the pool samples; data points: number of spots associated to an expression value for the locus; SD: standard deviation for the expression value expressed as percentage of the mean. Full results available as additional material (see text). *The segment window contains more than one gene, but the significance is assumed to be maintained because the expression value of this over- or under-expressed gene prevails over the others.

^a^Cytoband not available in Gene was derived from the UCSC Genome Browser [36].

^b^This gene, exceeding the limit of five genes for each list, has been shown for its relevance in the Discussion, being recurrent in other comparisons.

At chromosomal level, we calculated (in the TRAM "Chr" table) the median 'A'/'B' expression ratio for all the genes located in the same chromosome. The highest ratios were near to 1 (0.93 for chr22, 0.92 for chrX and chr21, 0.91 for chr19); other values were in the range from 0.90 (chr17 and chr12) to 0.76 (chrY).

We performed two additional transcriptome maps to investigate specifically sex-biased gene expression patterns (data not shown, results may be regenerated by the user by excluding/including or reimporting samples on the basis of data provided in Additional file 1): in particular, we compared male (pool 'A.1', n=11) vs. female DS AMKL cells (pool 'A.2', n=18). These datasets are derived from the samples for which the knowledge about the sex of the sample donor was available. The results showed a significant statistical correlation of data between male and female gene expression data (r=0.99, p-value<0.0001), showing a large overlap of results between the two transcriptome maps, with the exception of single genes with a well known sex-biased expression pattern. For example, *XIST*, which is specifically activated in female cells to start the X-inactivation process, turns out to be the most differentially expressed gene between female (value=402.60) and male (value=12.20) DS AMKL cells (ratio=33).

### Transcriptome map comparison of DS AMKL or non-DS AMKL vs. normal MK

Regional differential expression of pool 'A' (43 DS AMKL samples) or pool 'B' (45 non-DS AMKL samples) versus pool 'D' (19 normal MK cell samples, 411,381 data points) (Table 1) was investigated. An 'A'/'D' ratio value was determinable for 25,800 loci (Additional file 4). The main results are shown in Figure 1.

For what DS AMKL samples are concerned, results included 5 significantly differentially expressed segments in DS AMKL cells, 3 over- and 2 under-expressed (Table 2b). The highest expression ratio (20.81) between DS AMKL cells and normal MK was observed in the segment at coordinates 3,500,001-4,000,000 on chromosome 3, including the known gene *LRRN1*, encoding for a type I transmembrane protein. The second segment with highest expression ratio (18.07) was located on chromosome 1 (1q23) and contained *FAM78B* (family with sequence similarity 78, member B). The third segment was on chromosome 15 and included *NR2F2-AS1*, a non-coding RNA. The first significantly under-expressed segment (4q32.1) includes genes encoding for subunits of soluble guanylate cyclase (*GUCY1A3* and *GUCY1B3*), while the second spans the cluster of MK specific genes located on chromosome 4 (4q12-q21).

At single gene level, a fold increase higher than 18 was observed in all of the first 20 genes with the greatest expression ratios of DS AMKL vs. MK samples (Table 3b and Additional file 4). In particular, a 59-fold increase was observed for *TMEM241*, encoding a transmembrane protein of unknown function. Among the genes with the lowest 'A'/'D' expression ratio a 589.7-fold decrease was observed for *TSPAN10*, encoding for tetraspanin 10.

At chromosomal level, the highest ratio was observed for chr21 (1.75), the lowest (chr17), other values were in the range from 1.68 (chrY) to a value of 1.23 for chr17.

Regarding the non-DS AMKL samples, results obtained by default analysis and derived from 'B'/'D' expression ratio for 25,819 loci included one significantly over- and one significantly under-expressed segment (Table 2c). The highest expression ratio (22.30) between non-DS AMKL and normal MK was observed in the same segment on chr15, significantly over-expressed also in the DS AMKL transcriptome map. This segment was the only significantly over-expressed one in this comparison. Similarly, the only significantly under-expressed segment includes the cluster of MK specific genes on chromosome 4 also found to be under-expressed in DS AMKL samples (Table 2b).

At single gene level, a fold increase higher than 31 was observed in all of the first 20 genes with the greatest expression ratios of non-DS AMKL vs. MK samples (Table 3c and Additional file 5). In particular, a 156-fold increase was observed for *TMEM241*. Overall, there was a remarkable overlap between the most over- (*TMEM241*, *CMBL*, *ZNF445*, *SPRR4*) and under-expressed (*PF4V1*, *FLJ22184*, *FSIP2*, *PPP1R3B*, *HIST3H3*, *PIF1*, *SPSB4*, *ILDR1*, *MAP3K10*, *DRD4*, *LBX1*, *TSPAN10*) genes in DS and in non-DS AMKL samples (Additional files 4 and 5).

At chromosomal level, looking at the median 'B'/'D' expression ratio for the genes located in the same chromosome, the highest ratio was observed for chr21 (1.77) and chrY (1.73), followed by chr13 (1.71) and chr10 (1.59); other values were in the range from 1.58 (chr20), to a value of 1.24 (chrX).

### Transcriptome map comparison of DS AMKL or non DS AMKL vs. normal CB MK

**Figure 2:**
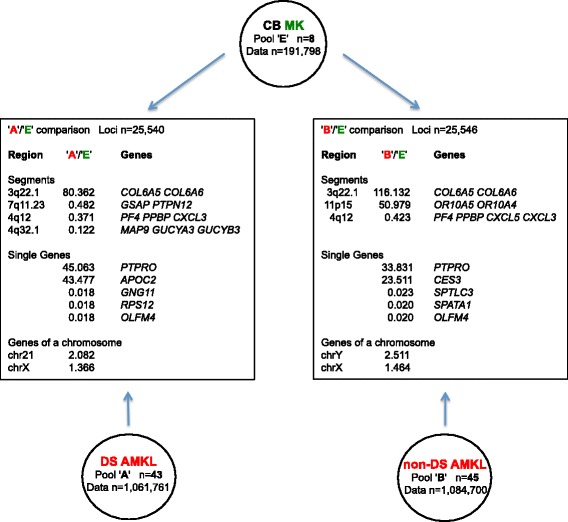
**Main results of DS AMKL vs. CB MK and non-DS AMKL vs. CB MK comparisons.** For each comparison the number of loci analyzed, the most over- or under-expressed segments and single genes, and the highest and lowest median expression ratios for all the genes located in the same chromosome are indicated.

Regional differential expression of pool 'A' (43 DS AMKL samples) or pool 'B' (45 non-DS AMKL samples) versus pool 'E' (8 normal cord blood (CB)-derived MK cell samples, 191,798 data points) (Table 1) was investigated. The main results are shown in Figure 2.

For what DS AMKL samples are concerned, results derived from 'A'/'E' expression ratio for 25,540 loci (Additional file 6) included 1 significantly over- and 3 under-expressed segments in DS AMKL cells (Table 4a). A remarkable expression ratio (80.36) between DS AMKL cells and normal CB MK was observed for a segment on chromosome 3 (3q22.1), including collagen-encoding *COL6A5* and *COL6A6* known loci. The three significantly under-expressed segments included the region on chromosome 4 (4q12-q21) with *PF4*, *PPBP* and *CXCL3* loci implied in MK differentiation.

**Table 4:** **Genomic segments significantly over- or under-expressed**

**a) DS AMKL vs. normal CB MK**					
**Chr and ** **location** ^**a**^	**Segment ** **start** ^**b**^	**Segment ** **end** ^**b**^	**'A'/'E' ratio**	**q-value**	**Genes in the segment** ^**c**^
**Over-expressed segments**					
chr3 3q22.1	130,000,001	130,500,000	80.36	0.00015	***COL6A5*** **+** ***COL6A6*** **+** Hs.596709+ **Hs.596805+** *PIK3R4*-
**Under-expressed segments**					
chr7 7q11.23	77,000,001	77,500,000	0.48	0.00117	***GSAP*** **-** ***PTPN12*** **-** LOC101059910- *RSBN1L-AS1*- *RSBN1L*- Hs.594486- **Hs.720279-** *TMEM60*- *PHTF2*-
chr4 4q12-q21	74,750,001	75,250,000	0.37	0.00119	***PF4*** **-** ***PPBP*** **-** *CXCL5*- ***CXCL3*** **-** *CXCL2*- *MTHFD2L*- Hs.662627- *EREG*-
chr4 4q32.1	156,250,001	156,750,000	0.12	0.00000	***MAP9*** **-** ***GUCY1A3*** **-** ***Hs.612374*** **-** ***GUCY1B3*** **-**
**b) Non-DS AMKL vs. normal CB MK**					
**Chr and location**	**Segment start**	**Segment end**	**'B'/'E' ratio**	**q-value**	**Genes in the segment**
**Over-expressed segments**					
chr3 3q22.1	130,000,001	130,500,000	116.13	0.00030	***COL6A5*** **+** ***COL6A6*** **+** Hs.596709+ **Hs.596805+** *PIK3R4*-
chr11 11p15	6,750,001	7,250,000	50.98	0.00117	*OR6A2*+ ***OR10A5*** **+** ***OR10A4*** **+** *ZNF215*- *ZNF214*- Hs.445849+ *NLRP14*+ *RBMXL2*- **LOC100506238+**
**Under-expressed segments**					
chr4 4q12-q21	74,750,001	75,250,000	0.42	0.00003	***PF4*** **-** ***PPBP*** **-** ***CXCL5*** **-** ***CXCL3*** **-** *CXCL2*- *MTHFD2L*- Hs.662627- *EREG*-

Data refer to the following comparisons: **a)** DS AMKL (pool 'A') vs. normal CB MK (pool 'E'); **b)** non-DS AMKL (pool 'B') vs. normal CB MK (pool 'E'). Analysis was performed using default parameters (see Methods section). Segments are sorted by increasing 'A'/'E' ratio in **a)**, 'B'/'E' ratio in **b)**. In the "Map" mode, TRAM displays UniGene EST clusters (with the prefix "Hs." in the case of *H. sapiens*) only if they have an expression value. Some segments are not shown for simplicity because they are over-lapping with those highlighted in one of the listed regions. The complete results for this model are available as on line additional material.

^a^Chr: chromosome. The segment location cytoband was derived from that of the first mapped gene within the segment.

^b^Segment Start/End: chromosomal coordinates for each segment.

^c^Bold and '+': over-expressed gene; bold and '-': under-expressed gene; '+' or '-': gene expression value higher or lower than the median value, respectively.

At single gene level, a fold increase higher than 15.7 was observed in all of the first 20 genes with the greatest expression ratios of DS AMKL cells vs. CB MK (Table 5a and Additional file 6). In particular, a 45-fold increase was observed for the tyrosine phosphatase receptor gene (*PTPRO*)*,* known to be involved in megakaryocytopoiesis.

**Table 5:** **List of the five most over- or under-expressed genes (all significantly, with q <0.05)**

**a) DS AMKL vs. normal CB MK**								
**Gene**	**Value 'A'**	**Value 'E'**	**Ratio 'A'/'E'**	**Location**	**Data points**		**SD**	**SD**
					**'A'**	**'E'**	**'A'**	**'E'**
**Over-expressed genes**								
*PTPRO*	679.7	15.1	45.063	12p13-p12	81	9	133.0	60.8
*APOC2* ^*a*^	431.7	9.9	43.477	19q13.2	54	10	81.4	43.4
*IFI27*	467.8	11.5	40.742	14q32	36	8	90.0	57.1
*HDC*	1,119.4	34.2	32.716	15q21-q22	36	8	98.8	168.1
*VIPR2* ^*ab*^	346.2	13.5	25.582	7q36.3	115	23	334.8	49.3
**Under-expressed genes**								
*CDKL1*	4.6	174.3	0.027	14q21.3	45	9	65.3	99.0
*ASAP2*	19.1	734.1	0.026	2p24	36	8	95.4	67.3
*GNG11*	18.9	1,042.3	0.018	7q21	36	8	119.7	81.2
*RPS12*	20.7	1,157.2	0.018	6q23.2	43	15	113.7	112.4
*OLFM4*	8.9	499.3	0.018	13q14.3	36	7	83.9	121.5
**b) Non-DS AMKL vs. normal CB MK**								
**Gene**	**Value 'B'**	**Value 'E'**	**Ratio 'B'/'E'**	**Location**	**Data points**		**SD**	**SD**
					**'B'**	**'E'**	**'B'**	**'E'**
**Over-expressed genes**								
*PTPRO* ^*b*^	510.3	15.1	33.831	12p13-p12	97	9	137.8	60.8
*CES3* ^*b*^	287.4	12.2	23.511	16q22.1	47	9	335.8	73.8
*SCD5* ^*ab*^	544.7	27.2	19.992	4q21.22	61	18	290.3	73.9
*TOP3B* ^*ab*^	150.2	7.5	19.922	22q11.22	60	14	431.8	19.7
*MT1E* ^*ab*^	237.1	11.9	19.899	16q13	45	8	92.5	27.3
**Under-expressed genes**								
Hs.23729^a^	2.3	85.1	0.027	1p13.2^c^	40	8	121.0	89.5
*PDE6C*	2.2	88.2	0.025	10q24	45	8	112.9	105.9
*SPTLC3*	4.8	203.7	0.023	20p12.1	47	9	86.5	110.7
*SPATA1*	7.0	356.2	0.020	1p22.3	40	7	127.4	77.9
*OLFM4*	9.7	499.3	0.020	13q14.3	45	7	105.1	121.5

Data refer to the following comparisons: **a)** DS AMKL (pool 'A') vs. normal CB MK (pool 'E'); **b)** non-DS AMKL (pool 'B') vs. normal CB MK (pool 'E'). Value: mean gene expression value normalized across all the pool samples; data points: number of spots associated to an expression value for the locus; 'SD': standard deviation for the expression value expressed as percentage of the mean. Full results available as additional material (see text).

^a^The segment window contains more than one gene, but the significance is assumed to be maintained because the expression value of this over- or under-expressed gene prevails over the others.

^b^According to the criteria detailed in Methods section, this gene is one of the five most over-expressed ones, but the value is not statistically significant due to the presence of a lot of loci associated with less than 5 data points in the CB MK integrated dataset.

^c^Cytoband not available in Gene was derived from the UCSC Genome Browser [36].

At chromosomal level, the highest ratio was observed for chr21 (2.08), followed by chrY (1.89); other values were in the range from 1.84 (chr22) to 1.37 (chrX).

Regarding the non-DS AMKL samples, results derived from 'B'/'E' expression ratio (for 25,546 loci) included 2 significantly over- and 1 under-expressed segments in non-DS AMKL (Table 4b). A remarkable expression ratio between non-DS AMKL and normal CB MK (116.13) was observed in the same segment on chromosome 3 (3q22.1), including collagen-encoding *COL6A5* and *COL6A6* known loci that was observed in DS AMKL samples. The second segment was specific of non-DS AMKL samples and included the two olfactive receptor genes *OR10A5* and *OR10A4*. The only significantly under-expressed segment included the region on chromosome 4 (4q12-q21) highly enriched in MK-specific loci (*PF4*, *PPBP*, *CXCL5* and *CXCL3*) as in the case of DS AMKL samples, and was extended to *PF4V1* locus.

At single gene level, a fold increase higher than 14.7 was observed in all of the first 20 genes with the greatest expression ratios of non-DS AMKL vs. CB MK samples (Table 5b and Additional file 7). In particular, a 33.8-fold increase was observed for *PTPRO,* encoding a tyrosine phosphatase receptor. Overall, there was some overlapping between the most over- and under-expressed genes in DS and in non-DS AMKL samples (Additional file 6 and Additional file 7).

At chromosomal level, regarding the median 'B'/'E' expression ratio for the genes located in the same chromosome, the highest ratio was observed for chrY (2.51), followed by chr21 (2.19); other values were in the range from 2.03 (chr20) to 1.46 (chrX).

### Transcriptome map comparison of DS AMKL vs. TMD

**Figure 3:**
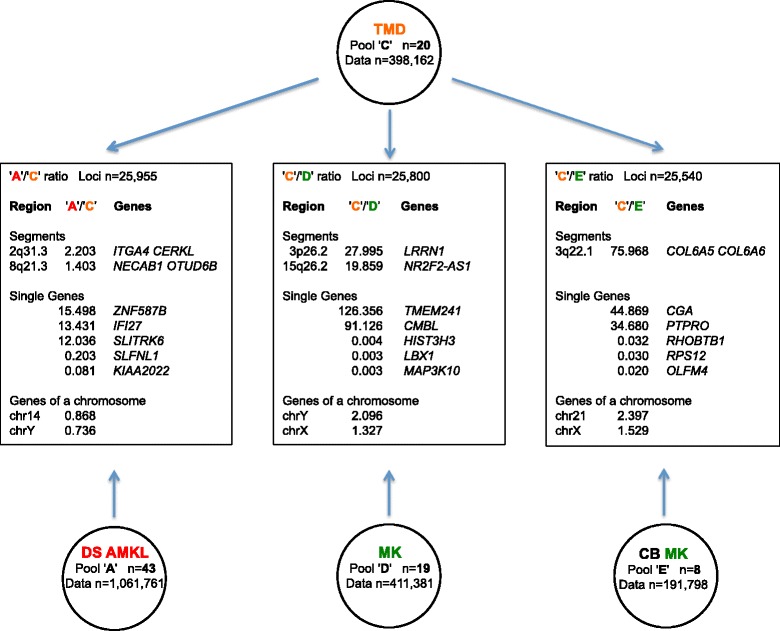
**Main results of DS AMKL vs. TMD, TMD vs. MK, TMD vs. CB MK comparisons.** For each comparison the number of loci analyzed, the most over- or under-expressed segments and single genes, and the highest and lowest median expression ratios for all the genes located in the same chromosome are indicated.

Regional differential expression of pool 'A' (43 DS AMKL samples) versus pool 'C' (20 TMD samples, 398,162 data points) (Table 1) was investigated. The main results are shown in Figure 3.

Results obtained by default analysis and derived from 'A'/'C' expression ratio for 25,955 loci (Additional file 8) included 2 significantly over-expressed segments in DS AMKL (Table 6a). The highest expression ratio (2.20) between DS AMKL and TMD was observed in a segment on chromosome 2 (2q31.3), including the known gene *ITGA4*, encoding an alpha 4 chain of integrin protein and *CERKL*, a gene responsible for retinitis pigmentosa and involved in the protection of cells from apoptosis induced by oxidative stress [47]. The second segment with the highest expression ratio (1.40) was located on chromosome 8 (8q21.3) and contained the *NECAB1* and *OTUD6B* genes, encoding for neuronal Ca(2+)-binding protein and the deubiquitinating enzyme, respectively.

**Table 6:** **Genomic segments significantly over- or under-expressed**

**a) DS AMKL vs. TMD**					
**Chr** ^**a**^ **and location**	**Segment ** **start** ^**b**^	**Segment ** **end** ^**b**^	**'A'/'C' ratio**	**q-value**	**Genes in the segment** ^**c**^
**Over-expressed segments**					
chr2 2q31.3	182,000,001	182,500,000	2.20	0.00045	***ITGA4*** **+** Hs.660611- Hs.658786+ ***CERKL+*** **Hs.72981+**
chr8 8q21.3	91,750,001	92,250,000	1.40	0.00051	***NECAB1*** **+** Hs.743640+ **LOC100127983+** *TMEM55A+ *LOC100506365- ***OTUD6B+*** *LRRC69*+
**b) TMD vs. normal MK**					
**Chr and location**	**Segment start**	**Segment end**	**'C'/'D' ratio**	**q-value**	**Genes in the segment**
**Over-expressed segments**					
chr3 3p26.2	3,500,001	4,000,000	28.00	0.00006	**Hs.241414+** ^**d**^ Hs.587205+ ***LRRN1*** **+ Hs.128128+**
chr15 15q26.2	96,750,001	97,250,000	19.86	0.00000	**Hs.677040+** ^**d**^ **Hs.661950+** ^**d**^ ***NR2F2-AS1*** **+** *NR2F2*- **Hs.592015+**
**c) TMD vs. normal CB MK**					
**Chr and location**	**Segment start**	**Segment end**	**'C'/'E' ratio**	**q-value**	**Genes in the segment**
chr3 3q22.1	130,000,001	130,500,000	75.97	0.00015	***COL6A5*** **+** ***COL6A6*** **+** Hs.596709+ **Hs.596805+** *PIK3R4*-

Data refer to the following comparisons: **a)** DS AMKL (pool 'A') vs. TMD (pool 'C'); **b)** TMD (pool 'C') vs. normal MK (pool 'D'); **c)** TMD (pool 'C') vs. normal CB MK (pool 'E'). Analysis was performed using default parameters (see Methods section). Segments are sorted by increasing 'A'/'C' ratio in **a)**, 'C'/'D' ratio in **b)** and 'C'/'E' ratio in **c)**. In the "Map" mode, TRAM displays UniGene EST clusters (with the prefix "Hs." in the case of *H. sapiens*) only if they have an expression value. Some segments are not shown for simplicity because they are over-lapping with those highlighted in one of the listed regions. The complete results for this model are available as additional material on line.

^a^Chr: chromosome. The segment location cytoband was derived from that of the first mapped gene within the segment.

^b^Segment Start/End: chromosomal coordinates for each segment.

^c^Bold and '+': over-expressed gene; bold and '-': under-expressed gene; '+' or '-': gene expression value higher or lower than the median value, respectively.

^d^This UniGene cluster contains EST with at least one Alu sequence, according to Repeat Masker [46]. For this reason, it can not be excluded that its over-expression is related to non-specific hybridization by Alu-containg probes.

At single gene level, a fold increase ranged from 15.5 to 3.5 for the first 20 genes with the greatest expression ratios of DS AMKL vs. TMD samples (Table 7a and Additional file 8). The highest fold increases were observed for the *ZNF587B* (15.5) and *IFI27* (13.4) genes, encoding for a zinc finger protein and the interferon alpha-inducible protein 27, respectively. The lowest 'A'/'C' expression ratios were observed for *KIAA2022* (10-fold decrease) and *SLFNL1* (5-fold decrease) genes.

**Table 7:** **List of the five genes most over- or under-expressed (all significantly, with q <0.05)**

**a) DS AMKL vs. TMD**								
**Gene**	**Value 'A'**	**Value 'C'**	**Ratio 'A'/'C'**	**Location**	**Data points**		**SD**	**SD**
					**'A'**	**'C'**	**'A'**	**'C'**
**Over-expressed genes**								
*ZNF587B*	615.0	39.7	15.498	19q13.43^c^	43	20	427.6	20.9
*IFI27*	467.8	34.8	13.431	14q32	36	11	90.0	91.4
*SLITRK6*	465.5	38.7	12.036	13q31.1	27	9	113.0	84.5
*BST2* ^*a*^	117.6	14.8	7.967	19p13.1	36	11	260.0	98.8
*ZNF521*	143.0	19.6	7.309	18q11.2	18	6	113.5	58.6
**Under-expressed genes**								
*ALS2CR12*	4.2	15.8	0.268	2q33.1	18	6	80.3	103.2
*GBP5*	11.6	43.8	0.266	1p22.2	18	6	75.7	110.6
*GHRH*	14.7	67.1	0.219	20q11.2	43	20	97.7	257.0
*SLFNL1* ^*a*^	18.9	92.9	0.203	1p34.2	18	6	90.3	122.1
*KIAA2022*	24.3	297.9	0.081	Xq13.3	27	9	52.2	260.9
**b) TMD vs. normal MK**								
**Gene**	**Value 'C'**	**Value 'D'**	**Ratio 'C'/'D'**	**Location**	**Data points**		**SD**	**SD**
					**'C'**	**'D'**	**'C'**	**'D'**
**Over-expressed genes**								
*TMEM241*	2,224.4	17.6	126.356	18q11.2	6	8	57.1	115.8
*CMBL*	1,063.5	11.7	91.126	5p15.2	9	8	168.8	30.1
*PTGS2*	2,256.4	59.2	38.145	1q25.2-q25.3	20	20	147.6	131.6
*CGA*	360.6	10.0	36.238	6q12-q21	14	19	159.7	27.3
*TMEFF2*	755.1	22.5	33.605	2q32.3	9	9	190.3	44.3
**Under-expressed genes**								
*ILDR1*	18.3	4,333.8	0.004	3q13.33	6	9	58.9	63.3
*DRD4*	9.3	2,327.6	0.004	11p15.5	11	19	52.3	135.2
*HIST3H3*	5.2	1,313.0	0.004	1q42	11	19	36.2	137.9
*LBX1*	4.3	1,358.5	0.003	10q24	11	19	26.6	152.6
*MAP3K10*	7.4	2,352.3	0.003	19q13.2	11	19	28.8	135.1
**c) TMD vs. normal CB MK**								
**Gene**	**Value 'C'**	**Value 'E'**	**Ratio 'C'/'E'**	**Location**	**Data points**		**SD**	**SD**
					**'C'**	**'E'**	**'C'**	**'E'**
**Over-expressed genes**								
*CGA*	360.6	8.0	44.869	6q12-q21	14	8	159.7	39.9
*PTPRO* ^*b*^	523.1	15.1	34.680	12p13-p12	25	9	116.8	60.8
*CLCA1* ^*b*^	319.2	9.9	32.380	1p22.3	20	8	116.7	17.5
*APOC2* ^*ab*^	301.6	9.9	30.372	19q13.2	17	10	66.6	43.4
*HDC* ^*ab*^	918.0	34.2	26.830	15q21-q22	11	8	59.9	168.1
**Under-expressed genes**								
*CDKL1*	5.9	174.3	0.034	14q21.3	14	9	82.0	99.0
*GNG11*	34.3	1,042.3	0.033	7q21	11	8	80.1	81.2
*RHOBTB1*	7.7	241.3	0.032	10q21.2	20	8	49.7	97.4
*RPS12*	34.6	1,157.2	0.030	6q23.2	20	15	77.4	112.4
*OLFM4*	10.0	499.3	0.020	13q14.3	11	7	56.0	121.5

Data refer to the following comparisons: **a)** DS AMKL (pool 'A') vs. TMD (pool 'C'); **b)** TMD (pool 'C') vs. normal MK (pool 'D'); **c)** TMD (pool 'C') vs. normal CB MK (pool 'E'). Value: mean gene expression value normalized across all the pool samples; data points: number of spots associated to an expression value for the locus; 'SD': standard deviation for the expression value expressed as percentage of the mean. Full results available as additional material (see text).

^a^The segment window contains more than one gene, but the significance is assumed to be maintained because the expression value of this over- or under-expressed gene prevails over the others.

^b^According to the criteria detailed in Methods section, this gene is one of the five most over-expressed ones, but the value is not statistically significant due to the presence of a lot of loci associated with less than 5 data points in the CB MK integrated dataset.

^c^Cytoband not available in Gene was derived from the UCSC Genome Browser [36].

At chromosomal level, the highest ratio was observed for chr14 (0.87, followed by 0.86 for chr5, chr21, chr8, chr12 and chr16), the lowest for chrY (0.74).

### Transcriptome map comparison of TMD vs. normal MK or CB MK cells

Regional differential expression of pool 'C' (20 TMD samples) versus pool 'D' (19 MK samples) or 'E' (8 CB MK samples) (Table 1) was investigated. The main results are shown in Figure 3.

For what MK samples are concerned, results obtained by default analysis and derived from 'C'/'D' expression ratios for 25,800 loci (Additional file 9) included 2 significantly over-expressed segments in TMD cells (Table 6b).

The highest expression ratio (28.0) between TMD and normal MK was observed in a segment on chromosome 3 including the known gene *LRRN1*, already observed as over-expressed in comparison of DS AMKL vs. normal MK (Table 2b). The second segment with the highest expression ratio (19.9) was located on chromosome 15, and contained the locus *NR2F2-AS1*, encoding for an antisense mRNA, already observed as over-expressed in comparison of DS AMKL and non-DS AMKL vs. normal MK (Table 2b and 2c).

At single gene level, the fold increase was higher than 16.5 for the first 20 genes with the greatest expression ratios (Table 7b and Additional file 9), with the highest fold increases for *TMEM241* (126.4) and the cysteine hydrolase gene (*CMBL*) (91.1). The lowest 'C'/'D' expression ratios were observed for a member of the serine/threonine kinase family (*MAP3K10*) and the homeobox gene (*LBX1*) (both with 333-fold decrease).

At chromosomal level, the highest ratio was observed for chrY (2.1) and chr21 (1.93), followed by chr13 (1.71), the lowest for chrX (1.33).

As far as the comparison of TMD with CB MK samples is concerned, results derived from the 'C'/'E' expression ratio for 25,540 loci (Additional file 10) included only 1 significantly over-expressed segment in TMD cells (Table 6c). The segment with a significant high expression ratio (76.0) between TMD and normal CB MK cells was on chromosome 3 (3q22.1), including the known genes *COL6A5* and *COL6A6* and already observed as over-expressed in DS as well in non-DS AMKL samples in comparison with CB MK samples.

At single gene level, a fold increase ranged from 44.9 to 15.1 for the first 20 genes with the greatest expression ratios (Table 7c and Additional file 10). The highest fold increases were observed for *CGA* (44.9), encoding for the alpha chain of the glycoprotein hormones and *PTPRO* (34.7), as already observed in non-DS AMKL vs. CB MK comparison. The lowest 'C'/'E' expression ratio was observed for *OLFM4* (50-fold decrease), encoding olfactomedin 4, an antiapoptotic factor that promotes tumor growth.

At chromosomal level, the highest ratio was observed for chr21 (2.40), followed by chrY (2.37) and chr20 (2.13), the lowest for chrX (1.53).

### Comparison with previously published data

As a result of the analysis above described, a reference integrated map for the expression of about 26,000 mapped sequences (~75% known genes and ~25% expression sequence tags - ESTs) was *de facto* generated for five cell types (DS AMKL cells, non-DS AMKL cells, TMD cells, MK and CB MK). This gave us the opportunity to compare our data with the expression values of specific known genes from previously published works about the considered cell types.

Following analysis of the main literature about AMKL, we selected 38 genes of interest and have tabulated their expression values desumed from our 8 differential maps, comparing these values to the ones previously described in different experimental settings (Table 8).

**Table 8:** **Comparison with previously published data**

**Gene**	**Location**	**References**	**'A'** **/'** **B'**	**'A'** **/**'**D'**	**'B'** **/**'**D'**	**'A** **'/'** **E'**	**'B'** **/'** **E'**	**'A'** **/'** **C'**	**'C'** **/** **'D'**	**'C** **'/'** **E'**
**MK markers**										
*BST2*	19p13.11	[23]	**0.53**	5.36	10.07	7.21	13.56	7.97	0.67	0.91
*GATA1*	Xp11.23	[13,22,48]	**3.39**	**1.45**	**0.43**	1.97	0.58	**1.19**	**1.22**	**1.66**
*GP1BA*	17p13.2	[43]	0.46	**0.07**	**0.14**	**0.25**	**0.53**	0.81	0.08	0.30
*HBG1*	11p15.5	[22]	**2.36**	1.40	0.59	1.07	0.45	0.78	1.79	1.37
*HBG2*	11p15.5	[22]	**2.47**	1.31	0.53	1.06	0.43	0.71	1.84	1.48
*ITGA2B*	17q21.32	[23]	0.82	**0.63**	0.77	1.52	1.84	1.05	0.60	1.44
*ITGB3*	17q21.32	[23]	0.75	**0.08**	**0.10**	**0.10**	**0.13**	0.65	0.12	0.15
*PF4*	4q12-q21	[25]	0.90	**0.06**	**0.06**	**0.11**	**0.12**	0.44	0.13	0.25
*PPBP*	4q12-q13	[25]	1.45	**0.10**	**0.07**	**0.13**	**0.09**	0.28	0.34	0.44
*TAL1*	1p32	[23]	**0.85**	**0.19**	**0.22**	**0.16**	**0.19**	1.05	0.18	0.16
**Chr21 genes**										
*ADAMTS1*	21q21.2	[49]	6.19	**7.02**	1.14	**7.14**	1.15	1.82	**3.85**	**3.91**
*BACH1*	21q22.11	[22]	**1.86**	1.44	0.77	1.04	0.56	1.55	0.93	0.67
*DYRK1A*	21q22.13	[50]	**1.61**	0.87	0.54	0.63	0.39	**1.14**	0.77	0.55
*ERG*	21q22.3	[18,22]	**0.68**	3.47	5.12	3.10	4.57	1.42	2.44	2.18
*ETS2*	21q22.2	[18,22]	**0.93**	1.11	1.20	1.39	1.49	0.73	1.52	1.90
*GABPA*	21q21.3	[18,22]	**1.39**	2.45	1.76	1.52	1.10	1.55	1.58	0.98
*RUNX1*	21q22.3	[18,22]	**0.71**	1.08	1.51	0.84	1.18	1.06	1.01	0.80
*SOD1*	21q22.11	[51]	**1.88**	1.52	0.81	2.78	1.48	1.52	1.00	1.84
*SON*	21q22.11	[22]	**1.28**	1.39	1.08	1.03	0.80	1.44	0.96	0.71
**Other genes**										
*APOC1*	19q13.2	[20,22,23]	**4.37**	10.04	2.30	16.26	3.72	**1.79**	5.63	9.11
*APOC2*	19q13.2	[22]	**4.36**	37.04	8.49	43.48	9.96	1.43	25.88	30.37
*APOE*	19q13.2	[22,23]	**2.18**	3.57	1.64	3.24	1.48	1.56	2.30	2.08
*CDA*	1p36.2-p35	[23]	**0.39**	0.40	1.03	0.88	2.24	0.88	0.46	1.00
*DICER1*	14q32.13	[18]	0.80	0.41	0.51	0.27	0.34	0.99	0.41	0.27
*FCER1A*	1q23	[22,23]	**6.32**	4.28	0.68	8.86	1.40	1.32	3.25	6.72
*GYPA*	4q31.21	[23]	**3.40**	1.56	0.46	0.89	0.26	1.19	1.31	0.75
*GYPB*	4q31.21	[23]	**2.81**	1.28	0.46	0.89	0.31	1.27	1.01	0.70
*GYPE*	4q31.1	[23]	**2.07**	1.63	0.79	1.71	0.82	1.72	0.95	0.99
*HDC*	15q21-q22	[22,23]	**17.10**	9.95	0.58	32.72	1.91	1.22	8.16	26.83
*IGF1R*	15q26.3	[17]	0.76	**1.18**	**1.55**	0.94	1.24	1.21	0.98	0.78
*ITGAL*	16p11.2	[52]	0.79	**0.21**	**0.26**	**0.09**	**0.11**	**1.02**	**0.20**	**0.09**
*KIT*	4q11-q12	[22]	**1.66**	3.34	2.01	3.08	1.85	1.22	2.74	2.53
*MPL*	1p34	[53]	1.11	**0.31**	**0.28**	0.22	0.20	1.23	**0.25**	0.18
*MTOR*	1p36.22	[17]	0.87	**1.57**	**1.82**	1.94	2.24	0.89	1.76	2.17
*MYCN*	2p24.3	[21]	0.35	1.00	2.87	0.77	2.19	**0.34**	2.99	2.28
*PRAME*	22q11.22	[21]	1.60	11.59	7.24	15.61	9.76	**4.18**	2.77	3.73
*SMOX*	20p13	[23]	**1.37**	0.76	0.55	1.02	0.75	0.79	0.96	1.30
*ST18*	8q11.23	[18]	0.90	0.74	0.82	0.74	0.82	0.78	0.96	0.95

Expression values of genes known for their role in MK differentiation and DS or non-DS AMKL development in each of the eight comparisons: DS AMKL (pool 'A') vs. non-DS AMKL (pool 'B'); DS AMKL (pool 'A') vs. normal MK (pool 'D'); non-DS AMKL (pool 'B') vs. normal MK (pool 'D'); DS AMKL (pool 'A') vs. normal CB MK (pool 'E') cells; non-DS AMKL (pool 'B') vs. normal CB MK (pool 'E'); DS AMKL (pool 'A') vs. TMD (pool 'C'); TMD (pool 'C') vs. normal MK (pool 'D'); TMD (pool 'C') vs. normal CB MK (pool 'E'). Data extracted from the full tables showing expression values and their ratio for about 17-26,000 loci for each comparison. In bold: expression ratio values are consistent with data available in the literature (see references indicated). The other values are reported for completeness. Descriptions of the gene symbols are given in Additional file 2.

The wide agreement of expression ratio values for specific genes between our data, generated by systematic meta-analysis of hundred of thousands of gene expression values from any gene expression profile available, and the data obtained by different marker-specific methods in published quantitative studies, is relevant for the validation of our maps that may so be used for exploring any other expression ratio in the considered biological conditions.

## Discussion

We have presented here a comprehensive analysis of transcriptome in human DS AMKL cells. Integration of data from different sources, including data obtained from different Authors using a variety of platforms, was made possible by a recent approach described by us for creation and analysis of transcriptome maps [25]. While most approaches are aimed to separate gene expression profiles related to the same biological source in subclasses, the TRAM tool provides means to integrate and summarize a pool of samples of the same biological origin leading to a global picture of gene expression for that condition. Moreover, TRAM identifies critical genomic regions and genes with significant differential expressions between two biological conditions.

Several Authors have determined gene expression profiles for DS or non-DS AMKL samples or have explicitly compared these two leukemic conditions. However, due to the rarity of the M7 subtype of leukemia and the need to limit the analysis to pediatric age because DS AMKL occurs almost exclusively in children, these studies were typically limited to small group of samples. In addition, most platforms used in the microarray studies are affected by omissions or errors in mapping a certain percentage of probes to specific loci in the genome. In our analysis, the use of a new version of the TRAM software (TRAM 1.1) allowed us to map thousands of previously uncharacterized microarray probes and to avoid the errors in probe assignment to human loci often present in the data supplied by the manufacturer along with the platforms.

Our data are derived from systematic integration of data from multiple sources at locus level (up-to-date rigorous assignment of each microarray probe to a specific human locus/transcript/EST cluster), map level (up-to-date fine mapping of each transcript on the genome map) and expression value level (assignment of a reference value to each locus in each cell type following an intra- and inter-sample normalization pipeline exploiting both parametric and non-parametric calculations). The combination of many gene expression profile datasets from different sources poses the problem of the batch effect, i.e. the systematic differences between batches (groups) of samples in microarray experiments due to purely technical reasons. However, the intrinsic resistance of the TRAM approach to the batch effect has been discussed previously [25], and it is indirectly confirmed by the clear biological meaning of the differential expression highlighted by the tool when comparison with previous direct key experimental knowledge is possible in several different types of tissues and organs [25,34,54].

A systematic comparison of AMKL originated from trisomy 21 cells versus non-trisomy 21 cells should highlight specific mechanisms [55] related to the presence of an extra copy of chr21 in DS children developing AMKL. Moreover, we presented a comparison with normal MK cells that has never been performed in other analyses about AMKL. Our global quantitative models of the transcriptome in the AMKL cells could also be useful to test hypotheses for correlations between any parameter associated to the condition (e.g., specific mutations or phenotype aspects) and specific changes in gene expression.

Our results, obtained in an integrated and open setting without any *a priori* assumption, show several previously unidentified aspects regarding specificity of AMKL originated by trisomy 21 cells.

First, there are only a few genomic regions significantly over- or under-expressed when comparing DS versus non-DS AMKL samples. This finding suggests that transcriptome maps of these two conditions are similar while on the other hand allows to focus to a small set of regions that appears to be critical in order to differentiate these disease conditions. Relevant differences regarding genes were reported in Table 2a (genomic segments) and Table 3a (single genes), with potential implications for the identification of diagnostic or therapeutic targets. There are three main regions over-expressed in DS AMKL vs. non-DS AMKL (Table 2a). The first one (15q21.2) contains *HDC* gene, whose mRNA is translated in the enzyme converting L-histidine to histamine produced by only a few cell types [56]; *HDC* mRNA increase has been shown to be associated to basophilic rather than to MK differentiation of pluripotent hematopoietic cells [57]. These observations led to the discovery of a skewing toward a potential basophilic differentiation for DS AMKL not highlighted in the original works from which the data were derived. Supporting this hypothesis, *FCER1A* mRNA, encoding the alpha subunit of the high-affinity IgE receptor, the initiator of the allergic response and strongly typical of basophilic differentiation, is 6.3 times over-expressed (the 8th most over-expressed known gene) in DS vs. non-DS AMKL, reinforcing the notion that in DS AMKL but not in non-DS AMKL the leukemic dedifferentiation involved the possibility of redirection toward basophilic differentiation. Remarkably, in [58] is demonstrated by an electron microscopy analysis that AMKL blast cells from children with DS may contain basophil-like granules which were almost totally absent in blasts from children with non-DS AMKL or adults with AMKL, so that our data allow to the visualization of the molecular correlation at the level of the whole transcriptome of a morphological feature observed more than 20 years ago. Two other genomic regions are over-expressed in DS vs. non-DS AMKL blasts. The first is the region of glycophorins genes (*GYPE*, *GYPA*, *GYPB*) on chr4, erythroid surface markers [59]: this reinforces the concept of a disturbance of multilineage myeloid hematopoiesis in DS AMKL and has been observed by flow cytometry in the non-neoplastic hematopoiesis itself in trisomy 21 [60]. The other is the region of apolipoproteins genes (*APOC1*, *APOC2*, *APOE*) on chr19 that has been described as a signature of progression from TMD to DS AMKL in a gene expression profile ([20], it was not possible to include this in our analysis), further underlining its specificity for DS vs. non-DS MK blast cells.

When grouping expression values by chromosome, the chromosome with the greatest global RNA output was chr21 in both TMD and DS AMKL vs. normal CB MK; we observed the same result in both DS and non-DS AMKL vs. normal MK comparisons. These data suggest that over-expression of chr21 genes is a key factor in AMKL development. In particular, *ADAMTS1*, encoding a protease known to inhibit angiogenesis [61] is the most over-expressed chr21 gene in DS vs. non-DS AMKL comparison. It is interesting to notice that this gene has been correlated to pediatric leukemias (ALL and DS AML) [49,62] but its exact role in leukemogenesis is still to be discovered. Our quantitative approach summarizing all values for each locus may clearly highlight mean expression ratio near to 1.5:1 for several chr21 genes when comparing DS AMKL vs. non-DS AMKL. This observation is consistent with the presence of an additional copy of the considered genes in trisomic cells. For example, *GABPA* expression presents a ratio close to 1.5:1 expected in trisomy of chr21, as well as other chr21 genes (*DYRK1A*, *SON, BACH1*) (Table 8). Even relatively small but significant differences (around 1.5-fold) in expression of numerous genes likely produce an aggregate effect, as observed in [63], where the same genes seem to be candidates to explain the impact of trisomy 21 in hematopoiesis abnormalities. For this reason it could be interesting to start from significant and robust meta-analysis data to plan functional approaches in the future. Moreover, our data are consistent with the previous observation that *RUNX1*, *ERG* and *ETS2* oncogenes, although located on chr21, are not over-expressed in DS vs. non-DS AMKL [18,22]. It has also been recently demonstrated that they are not located on a chr21 duplicated minimal region in two cases of AML of M0 subtype (FAB classification) [64]. As regard oncogenes, if chr21 oncogenes cited above appear not to be over-expressed in DS AMKL, *TRIB1* (chr8) may be a novel important oncogene for DS AMKL and its mutation is an earlier genetic event in leukemogenesis [65]. In particular, it has been shown that a mutation of *TRIB1* [65], a myeloid oncogene whose protein product is able to enhance ERK phosphorylation and to promote degradation of C/EBP family transcription factors, is a gain-of-function mutation remaining in leukocytes of the remission stage in which *GATA1* mutation disappeared. Our results show a mean value of 1.40 for the human *TRIB1* expression ratio between DS and non-DS AMKL samples, with a higher over-expression observed when comparing leukemic samples to normal MK (ratio 3.15 for DS AMKL and 2.26 for non-DS AMKL).

Although the expression of chr21 genes as a whole, or of some individual chr21 genes, may be coherent with the 3:2 gene expression ratio model in the comparison between trisomic and euploid cells, we note that discrepancies from this ideal model seen in our data for some of the comparisons we have made (Figures 1, 2 and 3) may be ascribed to the complexity of gene regulation in the aneuploid state [66,67], to individual variability [68] as well as to the general dysregulation typical of the neoplastic state for which specific cell types we analyze is concerned.

**Figure 4:**
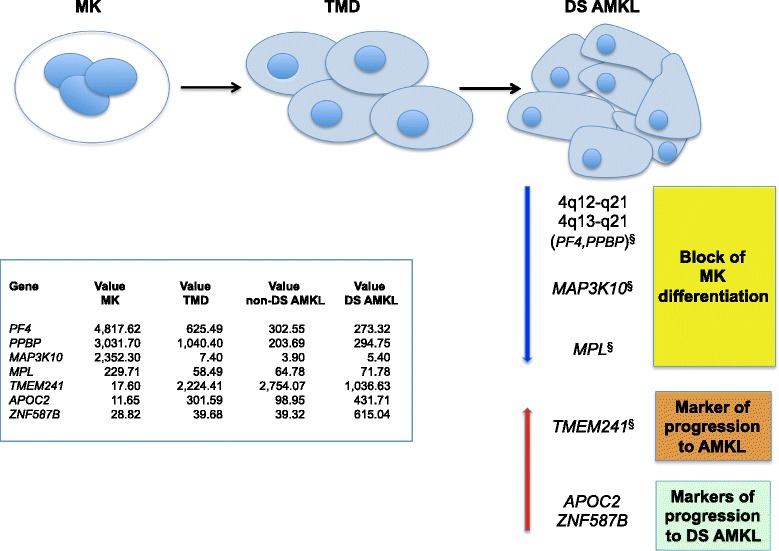
**Biological model of the transcriptome depicting progressive changes from MK to TMD then to DS AMKL.** Downward pointing arrow indicates the repression of genes involved in MK differentiation; upward pointing arrow indicates the over-expression of potential molecular markers of progression to AMKL. Value: mean gene expression value normalized across all the pool samples. ^§^Observed both in DS and non-DS AMKL.

Additional biologically relevant findings came from comparison of each type of megakaryoblastic leukemic condition with normal MK cells. Due to the role of the microenvironment in the hemopoiesis, including hemopoiesis in DS [69], it is expected that DS MK would present growth alterations due to trisomy 21 in both hemopoietic and microenvironment cells. From this point of view, DS MK cells would be the ideal control for the progression of a trisomic cell toward TMD and AMKL, however no gene expression profile dataset was available for this cell type. We propose here a biological model of the transcriptome depicting progressive changes from normal MK to TMD and then to DS AMKL, able to underline both shared and unique transcriptome map patterns for DS and non-DS variants of AMKL (Figure 4).

Noteworthy, the genomic segment on chr4 known to contain a cluster of genes highly specific for MK differentiation [25,70], was the highest significantly under-expressed segment in both DS and non-DS AMKL in comparison with normal MK. In particular, the more strongly under-expressed region 4q12-q21 contains a cluster of genes, including *PF4* (encoding for the platelet factor 4, a main component of platelet alpha granules) [71-73], and *PPBP* (encoding for beta-thromboglobulin) [70], that are the most up-regulated transcripts in the megakaryocytic differentiation from CD34+ hematopoietic progenitors [25]. This finding highlights a common final outcome of the block of MK cells differentiation in both DS and non-DS AMKL. It should be underlined that this result came from systematic *ab initio* analysis of more than 12,000 segments on the human genome including about 26,000 mapped loci, thus highlighting that this region critical for the MK differentiation is actually the more repressed in absolute when comparing transcriptome maps of AMKL (DS or non-DS) and normal MK cells.

In addition, the most under-expressed gene in TMD blasts when compared to normal MK cells is *MAP3K10*, encoding an activity of mitogen-activated protein kinase kinase kinase (MAPKKK). It is known that the mitogen-activated protein kinase (MAPK) pathway is involved in and is sufficient for megakaryocytic differentiation [74,75]. MAPK activity is present in several tens of human proteins, and we have identified the member *MAP3K10* as the critically repressed gene in the block of MK differentiation in the development of leukemia with MK features in that it appears down-regulated 300-fold in TMD cells and 500-fold in both DS and non-DS AMKL compared with normal MK samples (Tables 3 and 7). Finally, transcript for MPL, the receptor of thrombopoietin which is the primary regulator of normal thrombopoiesis (the formation of platelets) [53,73,76], is decreased by ~70% in either TMD, DS and non-DS AMKL cells vs. normal MK cells.

An exceedingly high over-expression of the gene located on chromosome 18 and encoding for the uncharacterized membrane protein TMEM241 has been found in both DS (59-fold) and non-DS (156-fold) AMKL cells vs. normal MK. Although this probe was not present in all considered experimental platforms, its extreme differential expression makes it a candidate for further studies as a marker of progression from normal MK to AMKL blasts, also due to its 126-fold over-expression in TMD vs. MK cells.

Moreover, we identified several signatures of progression specifically to DS AMKL. Remarkably, segments and genes up- or down-regulated in TMD in comparison with normal MK cells were highly similar to those specifically found in DS AMKL, underlining striking similarities between TMD and DS AMKL at the level of the whole transcriptome (already noted in [21], in their smaller set). On the other hand, a direct comparison between TMD and DS AMKL shows specific potential markers of progression to DS AMKL. As cited above, apolipoproteins genes (*APOC1*, *APOC2*, *APOE*) have been described as a signature of progression from TMD to DS AMKL [20] and it is interesting to notice that *APOC2* is among the 20 most expressed genes in the comparison between TMD and MK (25.88-fold increase), showing a progressive increase of expression from normal MK to TMD and then to DS AMKL. In our analysis, *ZNF587B* appears to be the most discriminant marker between TMD and DS AMKL. Again, this observation offers the opportunity to integrate and discuss single genes and pathways previously described as abnormally expressed in DS or non-DS AMKL (Table 8). For example, the *PRAME* gene, encoding for a tumor antigen [21] was identified as a specific marker for DS AMKL blasts (n=7), with no expression in TMD (n=9). While our meta-analysis on *PRAME* expression data points (36 for DS AMKL and 11 for TMD) confirmed a clear over-expression of *PRAME* in DS AMKL (4.2-fold increase, Table 8), it was not the most discriminant marker, that was exactly *ZNF587B*, while *PRAME* was the 33rd out of 25,955 transcripts ordered by decreasing DS AMKL vs. TMD expression ratio (Additional file 8).

Finally, since leukemias in infants or young children originate from fetal hematopoietic cells [17,18,26,27] and the progenitors (fetal/neonatal MKP) are present in the cord blood [28,29], comparisons with CB MK cells have been also performed. Data from DS and non-DS AMKL vs. CB MK comparisons confirmed the repression of the clusters of genes expressed in MK. The over-expression of a region with collagen genes emerged both in DS and non-DS AMKL as well as that of the single gene *PTPRO* (Table 5a and 5b), encoding for a tyrosine phosphatase receptor known to be involved in megakaryocytopoiesis and whose mRNA targeting by antisense oligonucleotides results in inhibited MK progenitor proliferation [77]. On the other hand, the difference between DS and non-DS AMKL vs. CB MK is shown by the over-expression of the two olfactive receptor genes *OR10A5* and *OR10A4* (Table 4) only in non-DS derived cells.

The analysis of enrichment in specific gene functions using the tool FuncAssociate, for the 100 most over- or under-expressed genes in the comparison of DS vs. non-DS AMKL and of both of them vs. MK cells gave no significant results, other than a significant enrichment in genes involved in sequence-specific DNA-binding in non-DS AMKL vs. MK cells (data not shown), highlighting the relevance of remodeling the transcription factor network in leukemia.

## Conclusions

Our results provide a systematic meta-analysis using any available gene expression profile dataset related to AMKL in pediatric age. These allow to identify more general trends and to produce a highly coherent view of the transcriptome depicting progressive changes from MK to TMD and then to DS AMKL. We believe that the originality of our results is due to several concurrent original features of the TRAM 1.1 platform. Advantages and relevant differences are: integration of the largest possible number of samples; integrated analysis of the largest possible number of genes (the integration of different platforms led us to assess expression ratio for about 26,000 loci, quantitating almost 4,000 genes in addition to the widely used platform U133A when used alone); absence of *a priori* filtering (in several works, this led to actual analysis of less than 50% of the genes present on the experimental platform); characterization at regional/map level in the study of gene expression (usually absent in the works from which data were obtained), relevant with regard to the study of an aneuploidy such as trisomy 21.

These results provide a new integrated model of the whole human transcriptome in DS and non DS AMKL, TMD and normal human MK cells, providing hints about pathophysiology of AMKL and also being useful to highlight possible clinical markers.

## Additional files

Additional file 1
**Table listing samples selected for the meta-analysis of gene expression profiles in DS AMKL (pool 'A'), non-DS AMKL (pool 'B'), TMD (pool 'C'), and MK (pool 'D' and 'E') cells.** All known data about patients whose samples were included in the meta-analysis are described. Additional file 2
**Table listing the genes cited in the paper.** We indicate gene symbol, gene name and location based on Gene database [35] or UCSC Genome Browser [36]. Additional file 3
**Table listing the 25,954 mapped loci of Transcriptome Map Comparison of DS vs. non-DS AMKL Cells (Pool 'A' vs Pool 'B') sorted in descending order of 'A'/'B' expression value.** N/A: not available. Additional file 4
**Table listing the 25,800 mapped loci of Transcriptome Map Comparison of DS AMKL vs. MK Cells (Pool 'A' vs Pool 'D') sorted in descending order of 'A'/'D' expression value.** N/A: not available. Additional file 5
**Table listing the 25,819 mapped loci of Transcriptome Map Comparison of non-DS AMKL vs. MK Cells (Pool 'B' vs Pool 'D') sorted in descending order of 'B'/'D' expression value.** N/A: not available. Additional file 6
**Table listing the 25,540 mapped loci of Transcriptome Map Comparison of DS AMKL vs. CB MK Cells (Pool 'A' vs Pool 'E') sorted in descending order of 'A'/'E' expression value.** N/A: not available. Additional file 7
**Table listing the 25,546 mapped loci of Transcriptome Map Comparison of non-DS AMKL vs. CB MK Cells (Pool 'B' vs Pool 'E') sorted in descending order of 'B'/'E' expression value.** N/A: not available. Additional file 8
**Table listing the 25,955 mapped loci of Transcriptome Map Comparison of DS AMKL vs. TMD Cells (Pool 'A' vs Pool 'C') sorted in descending order of 'A'/'C' expression value.** N/A: not available. Additional file 9
**Table listing the 25,800 mapped loci of Transcriptome Map Comparison of TMD vs. MK Cells (Pool 'C' vs Pool 'D') sorted in descending order of 'C'/'D' expression value.** N/A: not available. Additional file 10
**Table listing the 25,540 mapped loci of Transcriptome Map Comparison of TMD vs. CB MK Cells (Pool 'C' vs Pool 'E') sorted in descending order of 'C'/'E' expression value.** N/A: not available.

## Abbreviations

AMKL: Acute Megakaryoblastic Leukemia
DS: Down Syndrome
MK: Megakaryocytes
TMD: Transient Myeloproliferative Disorder
CB: Cord Blood
