# Supplementary material for: Integrated differential transcriptome maps of Acute Megakaryoblastic Leukemia (AMKL) in children with or without Down Syndrome (DS)
Source: BMC Med Genomics. 2014 Dec 5;7:63. doi: 10.1186/s12920-014-0063-z (PMC4304173; doi:10.1186/s12920-014-0063-z)
Supplement: Additional file 2 — Table listing the genes cited in the paper. We indicate gene symbol, gene name and location based on Gene database [35] or UCSC Genome Browser [36]. [file 12920_2014_63_MOESM2_ESM.pdf]

Additional file 2. List of the genes cited in the paper. We indicate gene symbol, gene name and location based on Gene database (http://www.ncbi.nlm.nih.gov/gene).

Gene symbol	Gene name	Location
ADAMTS1	ADAM metallopeptidase with thrombospondin type 1 motif, 1	21q21.2
ALAS1	aminolevulinate, delta-, synthase 1	3p21.1
ALS2CR12	amyotrophic lateral sclerosis 2 (juvenile) chromosome region, candidate 12	2q33.1
APOC1	apolipoprotein C-I	19q13.2
APOC2	apolipoprotein C-II	19q13.2
APOE	apolipoprotein E	19q13.2
ASAP2	ArfGAP with SH3 domain, ankyrin repeat and PH domain 2	2p24
ATP6V0D2	ATPase, H+ transporting, lysosomal 38kDa, V0 subunit d2	8q21.3*
BACH1	BTB and CNC homology 1, basic leucine zipper transcription factor 1	21q22.11
BST2	bone marrow stromal cell antigen 2	19p13.1
C5orf46	chromosome 5 open reading frame 46	5q32
CBS	cystathionine-beta-synthase	21q22.3
CD46	CD46 molecule, complement regulatory protein	1q32
CDA	cytidine deaminase	1p36.2-p35
CDKL1	cyclin-dependent kinase-like 1 (CDC2-related kinase)\	14q21.3
CDYL2	chromodomain protein, Y-like 2	16q23.2
CERKL	ceramide kinase-like	2q31.3
CES3	carboxylesterase 3	16q22.1
CGA	glycoprotein hormones, alpha polypeptide	6q12-q21
CLCA1	chloride channel accessory 1	1p22.3
CLEC12A	C-type lectin domain family 12, member A	12p13.2
CLEC2B	C-type lectin domain family 2, member B	12p13-p12
CMBL	carboxymethylenebutenolidase homolog (Pseudomonas)	5p15.2
COL6A5	collagen, type VI, alpha 5	3q22.1
COL6A6	collagen, type VI, alpha 6	3q22.1
CRTC1	CREB regulated transcription coactivator 1	19p13.11
CSRNP1	cysteine-serine-rich nuclear protein 1	3p22
CXCL3	chemokine (C-X-C motif) ligand 3	4q21
CXCL5	chemokine (C-X-C motif) ligand 5	4q13.3
DCK	deoxycytidine kinase	4q13.3-q21.1
DICER1	dicer 1, ribonuclease type III	14q32.13
DLGAP3	discs, large (Drosophila) homolog-associated protein 3	1p35.3-p34.1
DRD4	dopamine receptor D4	11p15.5
DYRK1A	dual-specificity tyrosine-(Y)-phosphorylation regulated kinase 1A	21q22.13
ERG	v-ets avian erythroblastosis virus E26 oncogene homolog	21q22.3
ETS2	v-ets avian erythroblastosis virus E26 oncogene homolog 2	21q22.2
FAM78B	family with sequence similarity 78, member B	1q24.1
FCER1A	Fc fragment of IgE, high affinity I, receptor for; alpha polypeptide	1q23
FLJ22184	putative uncharacterized protein FLJ22184	19p13.2

FSIP2	fibrous sheath interacting protein 2	2q32.1
GABPA	GA binding protein transcription factor, alpha subunit 60kDa	21q21.3
GATA1	GATA binding protein 1 (globin transcription factor 1)	Xp11.23
GATA2	GATA binding protein 2	3q21.3
GBP5	guanylate binding protein 5	1p22.2
GHRH	growth hormone releasing hormone	20q11.2
GNG11	guanine nucleotide binding protein (G protein), gamma 11	7q21
GP1BA	glycoprotein Ib (platelet), alpha polypeptide	17p13.2
GPR171	G protein-coupled receptor 171	3q25.1
GSAP	gamma-secretase activating protein	7q11.23
GYPA	glycophorin A (MNS blood group)	4q31.21
GYPB	glycophorin B (MNS blood group)	4q31.21
GYPE	glycophorin E (MNS blood group)	4q31.1
GUCY1A3	guanylate cyclase 1, soluble, alpha 3	4q31.1-q31.2
GUCY1B3	guanylate cyclase 1, soluble, beta 3	4q31.3-q33
HBG1	hemoglobin, gamma A	11p15.5
HBG2	hemoglobin, gamma G	11p15.5
HDC	histidine decarboxylase	15q21-q22
HIST3H3	histone cluster 3, H3	1q42
IFI27	interferon, alpha-inducible protein 27	14q32
IGF1R	insulin-like growth factor 1 receptor	15q26.3
ILDR1	immunoglobulin-like domain containing receptor 1	3q13.33
ITGA2B	integrin, alpha 2b (platelet glycoprotein IIb of IIb/IIIa complex, antigen CD41)	17q21.32
ITGA4	integrin, alpha 4 (antigen CD49D, alpha 4 subunit of VLA-4 receptor)	2q31.3
ITGAL	integrin, alpha L (antigen CD11A (p180), lymphocyte function-associated antigen 1; alpha polypeptide)	16p11.2
ITGB3	integrin, beta 3 (platelet glycoprotein IIIa, antigen CD61)	17q21.32
KIAA2022	KIAA2022	Xq13.3
KIT	v-kit Hardy-Zuckerman 4 feline sarcoma viral oncogene homolog	4q11-q12
KLRB1	killer cell lectin-like receptor subfamily B, member 1	12p13
KLRF1	killer cell lectin-like receptor subfamily F, member 1	12p13.31
LBX1	ladybird homeobox 1	10q24
LOC100287628	uncharacterized LOC100287628	16p13.2
LRRN1	leucine rich repeat neuronal 1	3p26.2
MAP3K10	mitogen-activated protein kinase kinase kinase 10	19q13.2
MAP9	microtubule-associated protein 9	4q32.1
MARCH2	membrane-associated ring finger (C3HC4) 2, E3 ubiquitin protein ligase	19p13.2
MED12L	mediator complex subunit 12-like	3q25.1
MPL	myeloproliferative leukemia virus oncogene	1p34
MPV17L	MPV17 mitochondrial membrane protein-like	16p13.11
MT1E	metallothionein 1E	16q13
MTOR	mechanistic target of rapamycin (serine/threonine kinase)	1p36.2
MYCN	v-myc myelocytomatosis viral related oncogene, neuroblastoma derived (avian)	2p24.3
NCOR2	nuclear receptor corepressor 2	12q24
NECAB1	N-terminal EF-hand calcium binding protein 1	8q21.3

NR2F2-AS1	NR2F2 antisense RNA 1	15q26.2
OLFM4	olfactomedin 4	13q14.3
OPHN1	oligophrenin 1	Xq12
OR10A4	olfactory receptor, family 10, subfamily A, member 4	11p15.4
OR10A5	olfactory receptor, family 10, subfamily A, member 5	11p15.4
OTUD6B	OTU domain containing 6B	8q21.3
P2RY13	purinergic receptor P2Y, G-protein coupled, 13	3q24
PDE6C	phosphodiesterase 6C, cGMP-specific, cone, alpha prime	10q24
PF4	platelet factor 4	4q12-q21
PF4V1	platelet factor 4 variant 1	4q12-q21
PIF1	PIF1 5'-to-3' DNA helicase	15q22.31
PPBP	pro-platelet basic protein (chemokine (C-X-C motif) ligand 7)	4q12-q13
PPP1R3B	protein phosphatase 1, regulatory subunit 3B	8p23.1
PRAME	preferentially expressed antigen in melanoma	22q11.22
PRF1	perforin 1 (pore forming protein)	10q22
PTGS2	prostaglandin-endoperoxide synthase 2 (prostaglandin G/H synthase and cyclooxygenase)	1q25.2-q25.3
PTPN12	protein tyrosine phosphatase, non-receptor type 12	7q11.23
PTPRO	protein tyrosine phosphatase, receptor type, O	12p13-p12
RHOBTB1	Rho-related BTB domain containing 1	10q21.2
RPS12	ribosomal protein S12	6q23.2
RUNX1	runt-related transcription factor 1	21q22.3
S100A8	S100 calcium binding protein A8	1q21
S100A9	S100 calcium binding protein A9	1q21
S100A12	S100 calcium binding protein A12	1q21
SCD5	stearoyl-CoA desaturase 5	4q21.22
SCGB3A2	secretoglobin, family 3A, member 2	5q32
SLFNL1	schlafen-like 1	1p34.2
SLITRK6	SLIT and NTRK-like family, member 6	13q31.1
SMOX	spermine oxidase	20p13
SOD1	superoxide dismutase 1, soluble	21q22.11
SON	SON DNA binding protein	21q22.11
SOSTDC1	sclerostin domain containing 1	7p21.1
SPATA1	spermatogenesis associated 1	1p22.3
SPINK2	serine peptidase inhibitor, Kazal type 2 (acrosin-trypsin inhibitor)	4q12
SPINK7	serine peptidase inhibitor, Kazal type 7 (putative)	5q32
SPRR4	small proline-rich protein 4	1q21.3
SPSB4	splA/ryanodine receptor domain and SOCS box containing 4	3q23
SPTLC3	serine palmitoyltransferase, long chain base subunit 3	20p12.1
ST18	suppression of tumorigenicity 18, zinc finger	8q11.23
TAL1	T-cell acute lymphocytic leukemia 1	1p32
TEK	TEK tyrosine kinase, endothelial	9p21
TERT	telomerase reverse transcriptase	5p15.33
TMEFF2	transmembrane protein with EGF-like and two follistatin-like domains 2	2q32.3
TMEM241	transmembrane protein 241	18q11.2

TNFSF14	tumor necrosis factor (ligand) superfamily, member 14	19p13.3
TOP3B	topoisomerase (DNA) III beta	22q11.22
TPSG1	tryptase gamma 1	16p13.3
TRIB1	tribbles pseudokinase 1	8q24.13
TSPAN10	tetraspanin 10	17q25.3
VIPR2	vasoactive intestinal peptide receptor 2	7q36.3
ZNF445	zinc finger protein 445	3p21.32
ZNF521	zinc finger protein 521	18q11.2
ZNF587B	zinc finger protein 587B	19q13.43*

* Cytoband was derived from the UCSC Genome Browser (http://genome-euro.ucsc.edu/cgi-bin/hgGateway).
